# Some Good and Some Bad: Sand Fly Salivary Proteins in the Control of Leishmaniasis and in Autoimmunity

**DOI:** 10.3389/fcimb.2022.839932

**Published:** 2022-02-25

**Authors:** Valeria Aoki, Maha Abdeladhim, Ning Li, Pedro Cecilio, Phillip Prisayanh, Luis A. Diaz, Jesus G. Valenzuela

**Affiliations:** ^1^ Department of Dermatology, Faculdade de Medicina da Universidade de São Paulo (FMUSP), Universidade de Sao Paulo, Sao Paulo, Brazil; ^2^ Vector Molecular Biology Section, Laboratory of Malaria and Vector Research, National Institute of Allergy and Infectious Diseases, National Institutes of Health, Rockville, MD, United States; ^3^ Department of Dermatology, University of North Carolina at Chapel Hill, Chapel Hill, NC, United States; ^4^ Vector Biology Section, Laboratory of Malaria and Vector Research, National Institute of Allergy and Infectious Diseases, National Institutes of Health, Rockville, MD, United States

**Keywords:** sand fly, salivary proteins, immunogenicity, cellular immunity, antibodies, autoimmunity

## Abstract

Sand flies are hematophagous insects responsible for the transmission of vector-borne diseases to humans. Prominent among these diseases is Leishmaniasis that affects the skin and mucous surfaces and organs such as liver and spleen. Importantly, the function of blood-sucking arthropods goes beyond merely transporting pathogens. The saliva of vectors of disease contains pharmacologically active components that facilitate blood feeding and often pathogen establishment. Transcriptomic and proteomic studies have enumerated the repertoire of sand fly salivary proteins and their potential use for the control of Leishmaniasis, either as biomarkers of vector exposure or as anti-*Leishmania* vaccines. However, a group of specific sand fly salivary proteins triggers formation of cross-reactive antibodies that bind the ectodomain of human desmoglein 1, a member of the epidermal desmosomal cadherins. These cross-reactive antibodies are associated with skin autoimmune blistering diseases, such as pemphigus, in certain immunogenetically predisposed individuals. In this review, we focus on two different aspects of sand fly salivary proteins in the context of human disease: The good, which refers to salivary proteins functioning as biomarkers of exposure or as anti-*Leishmania* vaccines, and the bad, which refers to salivary proteins as environmental triggers of autoimmune skin diseases.

## Introduction

Sand flies are phlebotomine arthropods and the main vectors of *Leishmania* parasites; sand flies are also relevant in other vector-borne diseases (VBDs) (Abdeladhim et al., 2014). Sand flies are distributed worldwide. They comprise six genera, two that are associated with human disease - *Phlebotomus* in the Old World (OW) and *Lutzomyia* in the New World (NW) (Akhoundi et al., 2016).

When a female sand fly takes a blood meal, it provokes skin damage that activates the hemostatic system (Ribeiro and Francischetti, 2003). Sand flies counteract host hemostasis system by injecting bioactive salivary components. These bioactive entities include potent vasodilators, e.g., maxadilan in *Lutzomiya longipalpis* (*Lu. longipalpis*), and adenosine in *Phlebotomus papatasi* (*P. papatasi*) sand flies (Lerner et al., 1991; Ribeiro et al., 1999), apyrases that inhibit platelet aggregation (Valenzuela et al., 2001; Anderson et al., 2006; Hamasaki et al., 2009), and inhibitors of the complement and coagulation cascades, e.g., lufaxin, a Factor Xa inhibitor, in *Lu. longipalpis* (Charlab et al., 1999; Collin et al., 2009; Abdeladhim et al., 2014). These agents are injected within small amounts of saliva to facilitate blood-feeding. The sand fly salivary proteome is composed of about 30 secreted proteins (Gomes and Oliveira, 2012) with quite diverse biological activities. Importantly, humans are constantly exposed to sand fly bites in disease endemic areas. Consequently, vector bites also have long-lasting systemic implications once sandfly salivary proteins become immunogenic.

Systemic immune responses to vector saliva are well documented. Brummer-Korvenkontio et al. reported antibody responses (IgG, IgG1, IgM, and IgE) to mosquito saliva in the NW (Brummer-Korvenkontio et al., 1994). Similarly, sera from children of endemic areas of Visceral Leishmaniasis (VL) and adults experimentally subjected to *Lu. longipalpis* bites recognized *Lu. longipalpis* salivary gland sonicate (SGS) with involvement of IgG (IgG1, and IgG4) and IgE antibodies (Gomes et al., 2002; Vinhas et al., 2007). Marzouki et al. reported the same IgG and IgE anti-SGS responses for the saliva of *P. papatasi* sand flies in endemic areas of Cutaneous Leishmaniasis (CL) (Marzouki et al., 2011). Importantly, cellular responses to sandfly saliva (particularly of pro-inflammatory nature, including IFN-γ recall responses) were equally detected in individuals pre-exposed to vector bites (Vinhas et al., 2007; Oliveira et al., 2013). Of note, at least until midlife, these individuals respond significantly to sand fly bites, which suggests lack of tolerization (Oliveira et al., 2013).

Sand fly salivary proteins may also act as environmental triggers of autoimmune diseases. A link between salivary proteins and autoimmunity is suggested by autoimmune blistering diseases, especially in endemic forms of pemphigus foliaceus (PF) (Diaz et al., 1989; Aoki et al., 2004). Pemphigus are organ-specific autoimmune skin diseases characterized by loss of epidermal adhesion (acantholysis) and blister formation (Lever, 1953; Amagai and Stanley, 2012). Endemic PF, also known as Fogo Selvagem (FS) shares with the sporadic nonendemic form of PF clinical features and pathogenic IgG4 autoantibodies (Rock et al., 1989) directed against the ectodomains of desmoglein 1 (Dsg1) (Amagai and Stanley, 2012). The IgG4 anti-Dsg1 autoantibody response is restricted to FS patients (Warren et al., 2003; Qaqish et al., 2009), whereas the non-pathogenic anti-Dsg1 IgG1 antibodies are detected in disease-free inhabitants of Brazilian endemic populations in the Limao Verde (LV) Amerindian reservation (Warren et al., 2000; Warren et al., 2003; Qaqish et al., 2009). Interestingly, non-pathogenic anti-Dsg1 antibodies are also detected in the sera of patients with Leishmaniasis and Chagas disease (Diaz et al., 2004). An isotype switch from IgG1 to IgG4 pathogenic anti-Dsg1 response may occur by the epitope spreading mechanism in individuals with the appropriate genetic HLA trait (Li et al., 2003). Notably, IgE and IgG4 anti-Dsg1 autoantibodies in FS patients cross-react with sand fly salivary proteins, likely because of antigenic mimicry (Qian et al., 2015; Qian et al., 2016; Diaz et al., 2020).

Although authors have systematized the knowledge derived from sand fly salivary proteins as disease-controlling agents (Rohousova and Volf, 2006; Andrade and Teixeira, 2012; Abdeladhim et al., 2014; Kamhawi et al., 2014), thus far, no review has included discussion of the participation of some of the sand fly salivary proteins as potential triggers of autoimmune disease. In this Mini Review, we offer an updated overview of sand fly salivary proteins in the context of human disease. The good news is that some proteins are markers of exposure and potential anti-*Leishmania* vaccines. The bad news is that some proteins may elicit autoimmunity.

## Markers of Exposure: Sand Fly Salivary Proteins as Tools for the Control of Leishmaniasis

The genomes of humans are remarkably alike; it is estimated that, at the DNA level, any two individuals share 99.9% identity (Collins and Mansoura, 2001). However, the 0.1% disparity is enough to condition significant inter-individual variances, including differences in immune responses (Kim-Hellmuth et al., 2017). Indeed, the composition and function of the human immune system are highly variable between healthy individuals, a consequence of heritable and non-heritable factors (Brodin and Davis, 2017). Therefore, it is not surprising that antibody responses vary immensely among humans, including responses to vaccination (Zimmermann and Curtis, 2019). Immunological diversity becomes quite relevant when we consider establishment of “markers of exposure” – essential tools for the determination of exposure to vector bites. Individuals exposed to vector bites show different patterns of antibody binding to salivary proteins (Gomes et al., 2002; Vinhas et al., 2007; Armiyanti et al., 2016). Some salivary proteins are recognized only by the sera of a few individuals. Other proteins are recognized by most sera, which makes these proteins near-universal markers of exposure. Importantly, such markers were proposed as strong indicators of the development of different VBDs (e.g., malaria and Lyme disease), and are important epidemiological risk-assessment tools (Schwartz et al., 1991; Remoue et al., 2006).

Sand flies are widely distributed; in the OW and NW, the genera *Phlebotomus* and *Lutzomyia* are responsible, respectively, for the transmission of *Leishmania* parasites (Akhoundi et al., 2016). In these regions there is an overlap of the (muco)cutaneous and visceral forms of Leishmaniasis, usually associated with different sandfly vectors, with significant disease burden (Akhoundi et al., 2016). Therefore, the development of markers that distinguish individuals exposed to different sand fly vectors is quite important from the epidemiological standpoint.

In the NW, particularly in Brazil, *Lu. intermedia*, and *Lu. longipalpis*, are the vectors for cutaneous and visceral Leishmaniasis, respectively (Bezerra et al., 2018). Two studies focused on this dichotomy in the search for markers of exposure. Teixeira et al. mined the salivary proteome of *Lu. longipalpis* in the quest for specific markers of exposure in the context of different hosts, including humans and dogs (Teixeira et al., 2010). Conversely, Carvalho et al. sought markers of exposure, particularly in humans, among the salivary proteome of *Lu. intermedia* (Carvalho et al., 2017). Teixeira et al. proposed LJM17, LJM11, and LJM111 (all yellow-related proteins; 45, 43, and 43 kDa, respectively) as potential markers of exposure to *Lu. Longipalpis* sand flies (Teixeira et al., 2010), whereas Carvalho et al. suggested LinB-13 (antigen 5-related protein; 28.4 kDa) as a potential marker of exposure to *Lu. intermedia* sand flies (Carvalho et al., 2017). LinB-13 was also deemed a potentially good disease biomarker (Carvalho et al., 2017). Of note, there was no cross-reactivity, which suggested that these proteins discriminate individuals exposed to each of these sand fly species, either alone, or in combination (LJM-17 + LJM-11), for better performance as markers (Souza et al., 2010; Teixeira et al., 2010; Carvalho et al., 2017).

In the OW a similar overlap is observed. *P. papatasi* sand flies, the main vectors of cutaneous Leishmaniasis are widely distributed around the Mediterranean basin, North Africa, throughout the Middle East and across the entire Indian subcontinent. In some *foci*, *P. papatasi* co-exists with *P. perniciosus* and *P. orientalis* sand flies, vectors of the causative agents of visceral Leishmaniasis, *Leishmania infantum* and *Leishmania donovani*, respectively (Akhoundi et al., 2016). Different studies have focused on the development of markers of exposure to help navigate such a complex epidemiological situation. In the context of CL, PpSP32, a silk-related protein was identified as the best marker of human exposure to the bites of *P. papatasi* sand flies. Cross-reactivity with salivary antigens from other co-endemic sand fly species was minimal, as demonstrated using the sera of dogs and humans exposed to *P. perniciosus* and *P. sergenti*, respectively (Marzouki et al., 2012; Marzouki et al., 2015; Mondragon-Shem et al., 2015). Importantly, a biomarker of exposure for dogs to the bites of *P. perniciosus* sand flies was also developed. PpeSP03B, a yellow-related protein was validated for the screening of dogs in *foci* of visceral Leishmaniasis caused by *L. infantum* parasites (Drahota et al., 2014; Kostalova et al., 2015; Kostalova et al., 2017; Willen et al., 2018; Willen et al., 2019). Additionally, two *P. orientalis* salivary proteins were identified as markers of exposure in humans - mAG5 (antigen 5-related protein) and mYEL1 (yellow-related protein) regarding visceral Leishmaniasis caused by *L. donovani* parasites (Sumova et al., 2018). Sima et al. proposed the same yellow-related protein (PorSP24 = mYEL1) as a suitable marker of exposure of domestic animals to the bites of *P. orientalis* sand flies (Sima et al., 2016).

## Sand Fly Salivary Proteins as Anti-*Leishmania* Vaccines

Sand fly saliva exacerbates the development of Leishmaniasis (Drahota et al., 2014; Marzouki et al., 2015; Mondragon-Shem et al., 2015; Kostalova et al., 2017). This aggravating effect is due to a combination of factors such as the bioactivity of the sand fly salivary proteins. Apart from preventing hemostasis, sand fly saliva/salivary proteins are immunomodulators. As reviewed elsewhere, sand fly salivary components can promote the generation of an anti-inflammatory *milieu via* different mechanisms. This anti-inflammatory condition is favorable for the persistence of *Leishmania*, and it modulates/impacts the recruitment/function of phagocytes essential for the survival of *Leishmania* in the host phagolysosome compartment (Collin et al., 2009; Abdeladhim et al., 2014). Therefore, immunization approaches based on sand fly salivary proteins have the potential to promote antibody-mediated inactivation of sand fly immunomodulatory components, thereby inhibiting establishment of infection. This immunization approach is exactly what was described in the context of two *Lu. longipalpis* salivary proteins, the hyaluronidase LuloHya (Charlab et al., 1999) and the endonuclease LJL138 (best known as Lundep) (Valenzuela et al., 2004). Immunization with each of these two proteins led to decreased pathology and parasite burden in mice infected with *L. major* parasites together with sandfly saliva; importantly, this phenotype was dependent of antibody responses because it was not observed in B-cell-deficient mice (Martin-Martin et al., 2018). Of note, Chagas et al. reported disease exacerbation mediated by LJL138 (Chagas et al., 2014), which suggested that the protective phenotype was a result of antibody-mediated protein inactivation (Martin-Martin et al., 2018). The same antibody-mediated blockage of activity can also explain the protection obtained against *L. major* infection in animals immunized with the *Lu. longipalpis* salivary protein LJL08 (maxadilan), although not exclusively because Th1 CD4+ T-cell-mediated responses seem also to have a function (Morris et al., 2001; Wheat et al., 2017). Still in this category, the blockage of the neutrophil chemoattractant activity of the yellow-related proteins PduM10 and PduM35 (Kato et al., 2006) also prevented the exacerbation effect of the saliva of *Phlebotomus duboscqi* sand flies in the context of a mouse model *L. major* infection (Guimaraes-Costa et al., 2021).

The antibody-mediated blockage of salivary protein activity may explain that naïve individuals, not previously exposed to sand fly bites or *Leishmania* parasites, display a higher risk of developing severe clinical forms of Leishmaniasis than non-naïve persons (Andrade et al., 2007). However, cell-mediated responses are probably the main contributors to such an epidemiological observation. Kamhawi et al. were first to show that pre-exposure to bites from noninfected sand flies induce protection against CL. This finding highlighted the crucial function of CD4+ T cell-dependent Th1 delayed-type hypersensitivity (DTH) responses (Kamhawi et al., 2000), which shaped the field of sand fly saliva-based anti-*Leishmania* vaccines. In most cases in which sand fly salivary proteins were proposed as anti-*Leishmania* vaccines, the choice was based on their potential to elicit DTH responses. Different animals were either pre-exposed to sand fly saliva followed by challenge with individual sandfly salivary proteins (*via* DNA vaccination) (Collin et al., 2009; Oliveira et al., 2015), or pre-immunized with DNA encoding individual sandfly salivary proteins and then challenged with sandfly saliva (Gomes et al., 2008; Oliveira et al., 2008; Xu et al., 2011; de Moura et al., 2013; Gholami et al., 2019). Only the proteins that induced significant DTH responses 48 h after challenge were deemed as potential vaccine candidates worthy of pre-clinical evaluation. This approach consistently led to the discovery of vaccines effective against different forms of Leishmaniasis in the context of vector transmission.

From the saliva of *Lu. longipalpis*, LJM-19 protected hamsters from fatal VL caused by *L. infantum* (Gomes et al., 2008) as well as in the context of cutaneous disease caused by *Leishmania braziliensis* (Tavares et al., 2011). The LJM-11 protein (from *Lu.longipalpis*) attenuated CL caused by *L. major* (and *L. braziliensis*) in mice (Xu et al., 2011) (Abi Abdallah et al., 2014; Cunha et al., 2018), as did LJL-14 (Cecilio et al., 2020). Notably, LJL-143 and LJM-17 were proposed as good vaccine candidates against canine Leishmaniasis caused by *L. infantum*, although an *in vivo* protective phenotype is yet to be demonstrated (Collin et al., 2009; Abbehusen et al., 2018). Additionally, from the saliva of the closely related *P. duboscqui* and *P. papatasi* sand flies, the homologous salivary proteins PpSP15 and PdSP15 (also known as PduM02) protected mice and non-human primates effectively from *L. major*-induced CL (Oliveira et al., 2008; Oliveira et al., 2015; Davarpanah et al., 2020). Three other proteins from the saliva of *P. papatasi*, PpSP36 (apyrase), PpSP42, and PpSP44 (both yellow-related proteins) were also proposed as good vaccine candidates for human CL (Tlili et al., 2018); however, efficacy results are either still missing, or contrary to this hypothesis in the case of PpSP44 in mice (Oliveira et al., 2008). Interestingly, another protein of the SP15 family, PsSP9 from the saliva of *P. sergenti* sand flies also protected mice from the development of CL caused by *L. tropica* (Gholami et al., 2019). Finally, from the saliva of *Lu. intermedia*, LinB-11 (SP13 family) conferred protection against cutaneous disease in a mouse model of *L. braziliensis* infection (de Moura et al., 2013). It is important to state that protection in the context of the aforesaid sand fly salivary antigens was associated with dominant pro-inflammatory (e.g. interferon-γ, and IL-12)/low anti-inflammatory (e.g. IL-4, IL-10, TGF-) CD4+ T cell-induced cytokine responses (Valenzuela et al., 2001; Gomes et al., 2008; Oliveira et al., 2008; Collin et al., 2009; Tavares et al., 2011; Xu et al., 2011; de Moura et al., 2013; Abi Abdallah et al., 2014; Oliveira et al., 2015; Abbehusen et al., 2018; Cunha et al., 2018; Tlili et al., 2018; Gholami et al., 2019; Cecilio et al., 2020; Davarpanah et al., 2020).

Detailed information of what is known and what is still missing on immune responses to sand fly salivary proteins including in the context of anti*-Leishmania* vaccines can be found in a few comprehensive reviews (Rohousova and Volf, 2006; Gomes and Oliveira, 2012). Of note, although these vector-derived antigens are effective individually as anti-*Leishmania* vaccines, their combination with *Leishmania*-derived antigens in several studies resulted in even more promising vaccine candidates (Zahedifard et al., 2014; Fiuza et al., 2016; Cecilio et al., 2017; Duthie et al., 2017; Fernandez et al., 2021). Considering that the natural infection caused by *Leishmania* is enhanced by some sand fly salivary proteins, the protective immune response would benefit from the combination of anti-*Leishmania* and anti-sand fly saliva responses.

## The Other Face of the Coin: Sandfly Salivary Proteins and Autoimmunity

Sand fly saliva is composed of a panoply of proteins with diverse functions. Some of these proteins are vaccine candidates or markers of disease exposure, whereas others can be pleiotropic and identified in both categories. Nevertheless, some markers of disease exposure are also identified as triggers of human autoimmunity, as observed in Fogo Selvagem, a blistering disease that targets Dsg1. Many studies on the etiology of FS were conducted in the Terena reservation of LV, ~1,600 individuals and a 3% prevalence for FS (Hans-Filho et al., 1996). FS patients produce IgG, IgM and IgE autoantibodies directed against Dsg1. IgG4 and IgG1 are the main IgG isotypes (Rock et al., 1989; Warren et al., 2003); IgG4 is pathogenic, as demonstrated in passive transfer mouse models (Rock et al., 1989; Evangelista et al., 2018) and the serum titers of IgG4 in patients correlate with disease activity (Warren et al., 2003; Li et al., 2003). In endemic areas, anti-Dsg1 IgG4 has a positive predictive value of 50% in identifying inhabitants with pre-clinical stages of FS (Qaqish et al., 2009). The IgG4 anti-Dsg1-restricted disease is strongly associated with HLADRB1*0102, 0404 and 1402 alleles, conferring a relative risk of 14 (Moraes et al., 1997).

Some rural populations in Brazil chronically exposed to insect bites, such as blackflies and reduviid (vector of Chagas disease) exhibit an autoantibody response against Dsg1 (Diaz et al., 2004).Interestingly, approximately 50% of the normal population possess nonpathogenic anti-Dsg1 autoantibodies (Warren et al., 2000; Qaqish et al., 2009). Epidemiological studies on the LV reservation strongly suggest that blood-feeding insects are risk factors for FS (Eaton et al., 1998; Aoki et al., 2004). Healthy individuals living in endemic areas of FS have higher frequency of IgM autoantibodies, compared with individuals from nonendemic FS regions, such as Japan and US. These IgM autoantibodies, although absent from the cord sera of mothers from LV (Hilario-Vargas et al., 2014), can be detected as early as five year of age (Diaz et al., 2008); the autoantibodies decrease as the inhabitants depart from endemic areas to urban sites, which suggests the influence of an environmental factor in autoantibody production (Diaz et al., 2008). Moreover, antigen selection is antigen driven even in pre-clinical stages, as demonstrated by our analysis of H and L chains of V genes of anti-Dsg1 IgM, reinforcing the idea of environmental triggers (Qian et al., 2009).

Recent advances in the characterization of Dsg1 epitopes show that 95% of IgG4 antibodies of FS sera recognize a 16-residue peptide (A_129_LNSMGQDLERPLELR_144_) located in the extracellular domain 1 of Dsg1 (Evangelista et al., 2018). This sequence overlaps the arginine-alanine-leucine (RAL) adhesive site of Dsg1, into which tryptophan residue 2 (Trp2) of desmocollin 1 (Dsc1) is inserted to bring desmosomal adhesion. The antigen-binding site of the FS IgG4 autoantibody binds a conformational epitope in the Dsg1 pocket. Mutation of M133, Q135, Q82 and V83 residues of the Dsg1 pocket abolish binding of FS IgG4 autoantibodies. Additionally, the Fab fragments of FS IgG4 autoantibodies inhibit the heterophilic aggregation of Dsg1/Dsc1 in a dose dependent manner (Evangelista et al., 2018). These studies strongly suggest that pathogenic FS IgG4 autoantibodies induce cell detachment and blisters in the epidermis by inhibiting the interaction of Dsg1 and Dsc1 desmosomal cadherins of FS patients. Steric hindrance and/or intracellular signaling or apoptosis are possible mechanisms under investigation.

**Figure 1:**
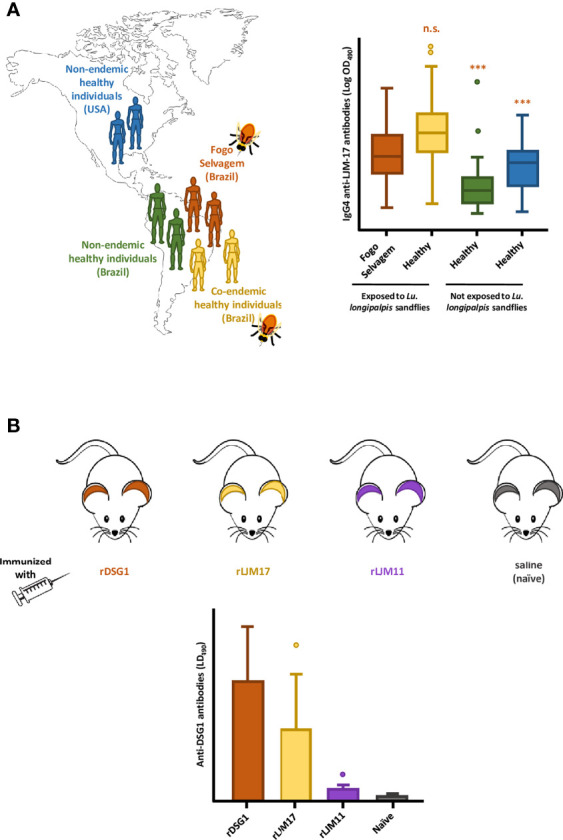
The potential association between the exposure to sandfly bites and the development of Fogo Selvagem (FS), in Limao Verde, Brazil. **(A)** In endemic areas of FS in Brazil, patients (Fogo Selvagem, orange) and healthy controls who are chronically exposed to the bites of *Lu. longipalpis* sandflies (Co-endemic healthy individuals, yellow) produce high and comparable levels of IgG4 antibodies against the sand fly salivary protein LJM17. This humoral immune response is not observed in normal individuals living in non-endemic areas, both in Brazil (Non-endemic healthy individuals, green), and in the USA (Non-endemic healthy individuals, blue). The relative levels of IGg4 antibodies anti- LJM17, are shown in the form of box-and-whiskers plots. **(B)** Mice immunized with recombinant LJM17 developed IgG1 antibodies (murine homologue of human IgG4) that cross-reacted with recombinant human Dsg1 (yellow). Mice in the positive and negative control groups, immunized with rDsg1 (orange) and saline (blue), respectively, showed the expected antibody responses against recombinant human Dsg1 (high, and very low, respectively. Additionally, mice immunized with LJM11 (purple) generated low titers of anti-Dsg1 antibodies. The levels of anti- Dsg1 antibodies are shown in the form bar graphs. This Figure is an adaptation of the data published by Diaz et al. (2020). ***(p< 0.001), n.s., normal human sera.

In Brazil, FS endemic sites overlap with areas of high prevalence of VBDs, especially Leishmaniasis (Diaz et al., 1989). Circulating anti-Dsg1 autoantibodies are detected in patients with insect-borne diseases such as Leishmaniasis and Chagas disease (Diaz et al., 2004; Walsh et al., 2017) and also in dogs and cats (Ginel et al., 1993). We then hypothesized that chronic exposure to insect bites and the salivary antigens therein could be a relevant trigger to FS. To understand whether the chronic exposure to insect bites (or insect salivary antigens) is a relevant trigger to FS, we collected serum samples from FS patients and investigated their reactivity toward *Lu. longipalpis* SGH (Valenzuela et al., 2004; Xu et al., 2011; Abdeladhim et al., 2014). We found significant correlation between levels of IgG4 and anti-IgE antibodies directed against *Lu. longipalpis* LJM 17 and 11 with anti-Dsg1 autoantibodies due to possible cross-reactivity (Qian et al., 2012; Qian et al., 2015). Further studies showed that sera from healthy controls and FS patients from endemic sites exhibited significant higher levels of IgG4 anti-LJM17 antibodies compared to nonendemic controls. Moreover, IgG anti-Dsg1 and IgG4 anti-LJM17 and anti-LJM11 antibodies positively correlated in normal settlers and FS patients (Diaz et al., 2020) (**Figure 1A**). Mice immunized with recombinant LJM17 developed nonpathogenic IgG1 antibodies (murine homologous of human IgG4) that cross-reacted with recombinant human Dsg1 (**Figure 1B**). We also identified short-sequence homologies of surface-exposed residues within the human DSG1 ectodomain and LJM17 (Diaz et al., 2020).

In the OW, Tunisians with endemic PF (Bastuji-Garin et al., 1995; Zaraa et al., 2012) have an increased IgG4 antibody response to *P. papatasi* salivary proteins, particularly SP32 (Marzouki et al., 2011; Marzouki et al., 2015; Marzouki et al., 2020). Marzouki et al. showed that PpSP32 bound directly to Dsg1 and Dsg3 forming immunogenic complexes; however, mice immunized with PpSP32 developed non-cross-reactive antibodies that recognized Dsg1 and Dsg3 (Marzouki et al., 2020). Marzouki et al. (2020) suggested that the PpSP32/Dsg1 and PpSP32/Dsg3 complexes induce loss of tolerance to these autoantigens and trigger pemphigus in genetically predisposed individuals.

Altogether, studies in different geographical settings suggest an association between the exposure of pre-disposed individuals to sand fly bites, and the development of autoimmune blistering diseases. The potential cross-reactivity of some sand fly salivary gland proteins (LJM 17 and 11 in the NW and PpSP32 in the OW) with Dsg1, the autoantigen of endemic pemphigus foliaceus, indicates the need for a careful choice when selecting such proteins as candidates for anti-*Leishmania* vaccines.

## Concluding Remarks

**Figure 2:**
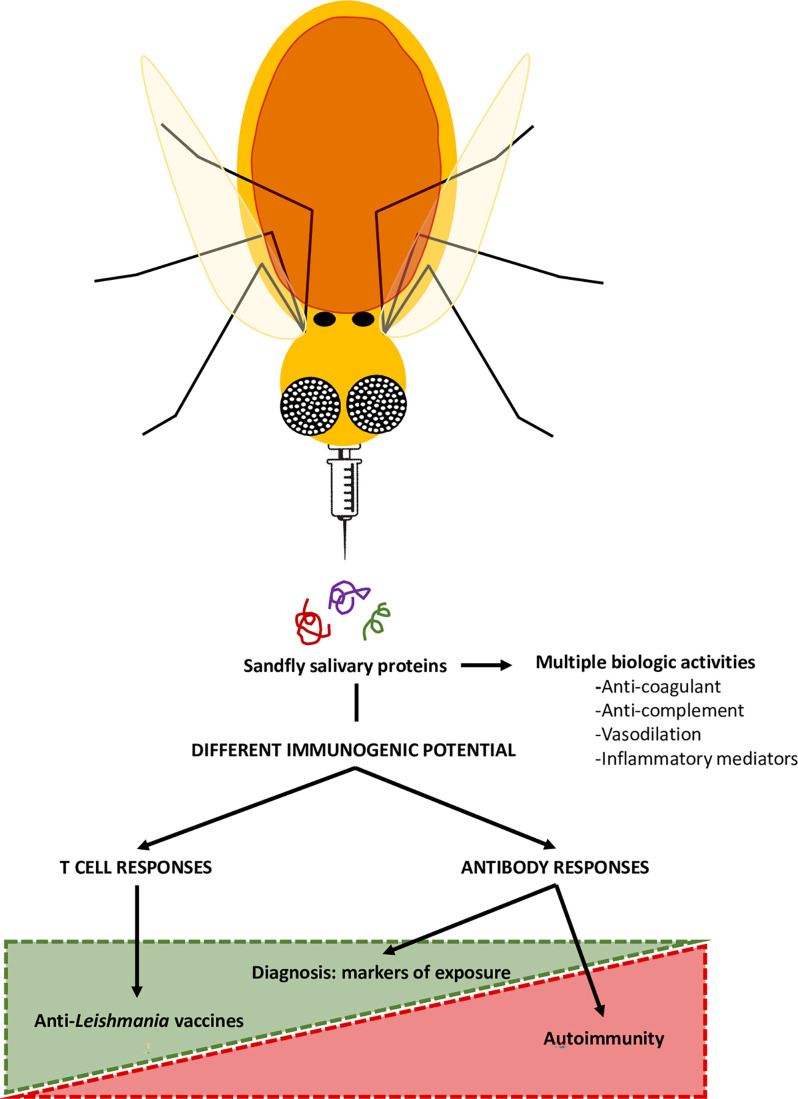
Sand fly salivary proteins in the control of Leishmaniasis and in autoimmunity. The saliva of blood-sucking arthropods, including sandflies, here represented as flying needles, contains components with immunomodulatory and anti-hemostatic properties. However, these proteins are also immunogenic, and, thus able to induce systemic immune responses. Therefore, some proteins may be used as markers of exposure of sandfly bites, with epidemiological value, or as components of anti-*Leishmania* vaccines. However, certain sand fly salivary proteins can sensitize the host and potentially trigger the formation of cross-reactive antibodies that may lead to the development of autoimmune blistering diseases, such as pemphigus foliaceus.

The birth of transcriptomics and proteomics allowed the detailed analysis of the salivary proteins of different sand fly species, especially in the field of infectious diseases. Some molecules were proposed as markers of exposure in endemic areas of *Leishmaniasis*, whilst others were defined as promising anti-*Leishmania* vaccine candidates; however, some are potential environmental triggers of autoimmune skin diseases (**Figure 2**). **Table 1** depicts a summary of sand fly salivary proteins and their potential role as markers of exposure, vaccine components or triggers in autoimmunity. Importantly, this tool or trigger duality must be patent in the development of sand fly saliva based anti*-Leishmania* vaccines, and only those molecules which are not inducers of autoimmunity responses (auspiciously most of the salivary gland proteins) should be applied for clinical development studies.

**Table 1:** Sand fly salivary proteins as markers of exposure, anti-*Leishmania* vaccines, and potential triggers of autoimmunity.

	Sand fly species	Salivary Protein	Salivary Protein family	Species tested	Ref.
**Markers of exposure**	*Lu. longipalpis*	LJM11	Yellow-related protein	Humans, dogs, chicken	(Teixeira et al., 2010)
		LJM17	Yellow-related protein	Humans, dogs, chicken, foxes	(Teixeira et al., 2010)
		LJM111	Yellow-related protein	Humans	(Teixeira et al., 2010)
	*Lu. intermedia*	Linb-13	Antigen-5-related protein	Humans	(Carvalho et al., 2017)
	*P. papatasi*	PpSP32	Silk-related protein	Humans	(Marzouki et al., 2012; Marzouki et al., 2015; Mondragon-Shem et al., 2015)
	*P. perniciosus*	PpeP03B	Yellow-related protein	Dogs	(Drahota et al., 2014; Kostalova et al., 2015; Kostalova et al., 2017; Willen et al., 2018; Willen et al., 2019)
	*P. orientalis*	mAG5	Antigen-5-related protein	Humans	(Sumova et al., 2018)
		mYEL1 or PorSP24	Yellow-related protein	Humans, domestic animals	(Sima et al., 2016; Sumova et al., 2018)
**Anti-*Leishmania* vaccines**	*Lu. Longipalpis*	LJM-19	SALO	Hamsters	(Gomes et al., 2008; Tavares et al., 2011)
		LJM11	Yellow-related protein	Mice	(Xu et al., 2011; Abi Abdallah et al., 2014; Cunha et al., 2018)
		LJM17	Yellow-related protein	Dogs	(Collin et al., 2009; Abbehusen et al., 2018)
		LJL143	Lufaxin	Dogs	(Collin et al., 2009; Abbehusen et al., 2018)
	*Lu. Intermedia*	Linb-11	SP13 family	Mice	(de Moura et al., 2013)
	*P. papatasi*	PpSP15	OBP-related protein	Mice	(Oliveira et al., 2008; Davarpanah et al., 2020)
		PpSP36	Apyrase	Humans	(Tlili et al., 2018)
		PpSP42	Yellow-related protein	Humans	(Tlili et al., 2018)
		PpSP44	Yellow-related protein	Humans	(Tlili et al., 2018)
	*P. duboscqi*	PdSP15 (PduM02)	OBP-related protein	Non-Human primates	(Oliveira et al., 2015)
	*P. sergenti*	PsSP9	OBP-related protein	Mice	(Gholami et al., 2019)
**Potential triggers of autoimmunity**	*Lu. longipalpis*	LJM11	Yellow-related protein	/	(Diaz et al., 2020)
		LJM17	Yellow-related protein	/	(Diaz et al., 2020)
	*P. papatasi*	PpSP32	Silk-related protein	/	(Zaraa et al., 2012)

## Ethics Statement

The human studies performed in this investigation were approved by Institutional Review Boards from the University of North Carolina and the University of Sao Paulo.

## Author Contributions

All the authors meet all criteria for authorship in the ICMJE recommendations. All authors were involved in the conceptualization, data acquisition, interpretation of data, and writing this minireview. All Authors approved the final submitted version. All the authors agreed to be accountable for all aspects of the work.

## Funding

This research was supported in part by RO1 AR32599 and CTSA-UL1TR002489 (LAD) and the Intramural Research Programs at the National Institute of Allergy and Infectious Diseases, National Institutes of Health (JV, MA, PC).

## Conflict of Interest

The authors declare that the research was conducted in the absence of any commercial or financial relationships that could be construed as a potential conflict of interest.

The handling Editor declared a past co-authorship with one of the authors JV.

## Publisher’s Note

All claims expressed in this article are solely those of the authors and do not necessarily represent those of their affiliated organizations, or those of the publisher, the editors and the reviewers. Any product that may be evaluated in this article, or claim that may be made by its manufacturer, is not guaranteed or endorsed by the publisher.
